# Odor‐induced changes in the connectivity of the olfactory network in individuals with subjective cognitive decline

**DOI:** 10.1002/alz70857_101542

**Published:** 2025-12-24

**Authors:** Yajing Zhu, Bing Zhang

**Affiliations:** ^1^ Nanjing Drum Tower Hospital, Affiliated Hospital Clinical College of Nanjing Medical University, Nanjing, Jiangsu, China

## Abstract

**Background:**

Patients with Alzheimer's disease (AD) exhibit olfactory impairments in the early stages of the disease. Individuals with subjective cognitive decline (SCD) are at high risk of preclinical AD. However, it is unclear whether individuals with SCD have impaired olfactory function, and the underlying neural mechanisms remain unknown.

**Method:**

A total of 61 normal controls (NC) and 63 individuals with SCD were included. All participants were assessed with cognitive scale assessments, olfactory behavioral tests, and olfactory task‐based functional magnetic resonance imaging (fMRI) examinations. Based on the literature, two olfactory brain networks were defined: the primary olfactory brain network and the advanced olfactory brain network. The primary olfactory brain network included the bilateral piriform cortex, amygdala, and entorhinal cortex. The advanced olfactory brain network included bilateral hippocampus, insula cortex, and orbitofrontal cortex. A general linear model was used to calculate functional connectivity within and between networks under odor stimulation. Additionally, correlations between functional connectivity and cognitive performance as well as olfactory behavioral performance were calculated, using gender, age, and years of education as covariates.

**Result:**

The olfactory identification scores and SCD‐Q scale scores of the SCD group were significantly higher than those of the NC group. Under odor stimulation, the SCD group showed significantly lower connectivity between the right entorhinal cortex and the left insular cortex compared to the NC group, while connectivity between the left piriform cortex and the left amygdala, the left piriform cortex and the right hippocampus, and the right insular cortex and the left orbitofrontal cortex were significantly higher in the SCD group than in the NC group (FDR corrected, *p* < 0.05). The altered functional connectivity in the SCD group was significantly positively correlated with olfactory thresholds, identification, and memory functions.

**Conclusion:**

The results indicate that in response to odor stimulation, individuals with SCD primarily exhibit enhanced functional connectivity in key regions within the olfactory brain network, which may represent a potential neural mechanism for maintaining normal olfactory perception.

